# Wnt controls the medial–lateral subdivision of the *Drosophila* head

**DOI:** 10.1098/rsbl.2018.0258

**Published:** 2018-07-25

**Authors:** M. S. Magri, M. A. Domínguez-Cejudo, F. Casares

**Affiliations:** GEM-DMC2 María de Maeztu Unit of Excellence, The CABD (CSIC-UPO-JA), 41013 Seville, Spain

**Keywords:** Wnt, *Drosophila*, insect head

## Abstract

In insects, the subdivision of the head into a lateral region, harbouring the compound eyes (CEs), and a dorsal (medial) region, where the ocelli localize, is conserved. This organization might have been already present in the insects' euarthropodan ancestors. In *Drosophila,* the Wnt-1 homologue *wingless (wg)* plays a major role in the genetic subdivision of the head. To analyse specifically the role of *wg* signalling in the development of the dorsal head, we attenuated this pathway specifically in this region by genetic means. We find that loss of *wg* signalling transforms the dorsal/medial head into lateral head structures, including the development of ectopic CEs*.* Our genetic analysis further suggests that *wg* signalling organizes the dorsal head medial–lateral axis by controlling, at least in part, the expression domains of the transcription factors Otd and Ey/Pax6.

## 1. Introduction

Within insects, the structure of the adult head is essentially conserved, despite the multiple morphological and functional specializations of its mouthparts and sensory organs [1–3]. In particular, the subdivision of the head into a lateral region, where the compound eyes (CEs) are located, and a dorsal/medial region, the ‘head vertex’, harbouring the ocelli (ocellar complex, OC) was present already in Cambrian euarthropods [4]. Still, the genetic mechanisms responsible for this subdivision are not well understood. In *Drosophila,* the Wnt gene wingless (*wg*) plays major roles in head development, including limiting the length of the signalling centre that induces retina development [5–10]. However, *wg* expression is dynamic and most studies have made use of *wg* mutant alleles, which make it difficult to determine what role its signalling plays in the dorsal head, where ocelli develop. Here, we have addressed this role by targeting the *wg* signalling pathway specifically in this region.

## 2. Results

### (a) Attenuation of the Wnt-canonical pathway in the prospective medial head capsule results in its transformation into lateral head structures

**Figure 1.**
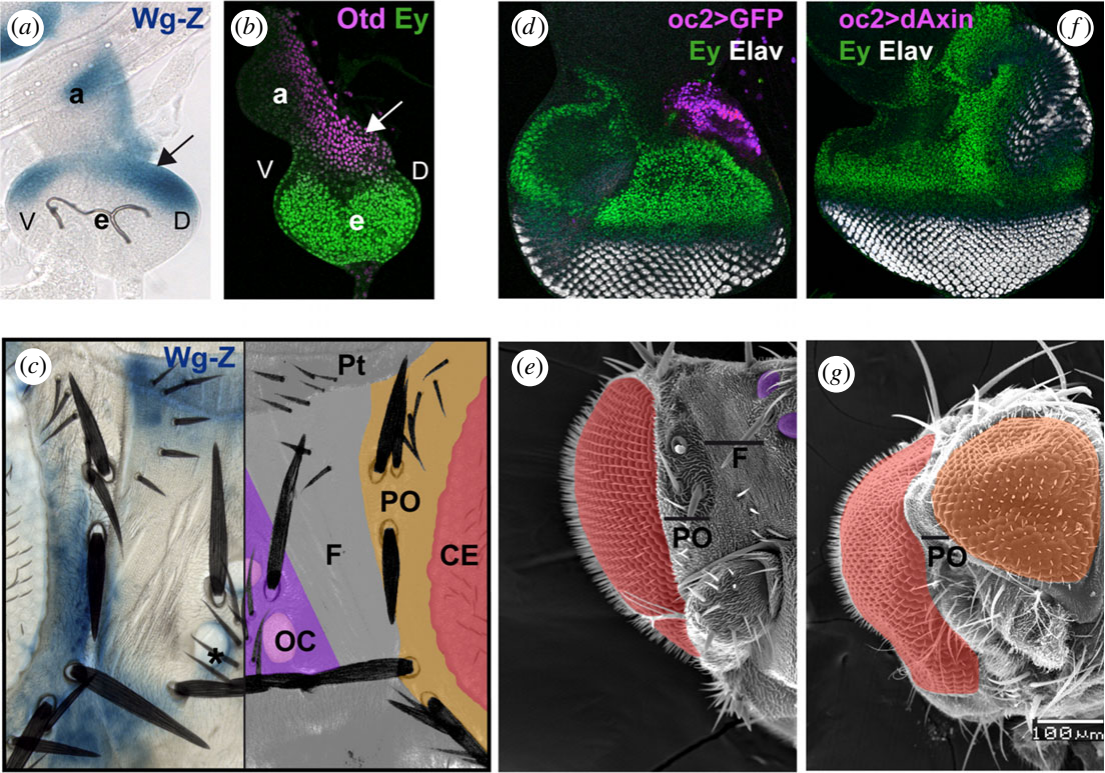
Attenuating the wg/Wnt signalling pathway results in the medial to lateral transformation of the *Drosophila* head. (*a,b*) Early third larva stage discs. (*a*) X-gal staining of the *wg* transcriptional reporter *wg-Z.* (*b*) Disc co-stained with anti-Otd (magenta) and anti-Ey (green) antibodies, showing complementary expression domains. The arrow in (*a*) and (*b*) points to the prospective dorsal head region, where ocelli develop. (*c*) X-Gal-stained *wg-Z* adult head (left) and schematic representation of dorsal head regions (right). *wg* expression is detected in the periocular cuticle (PO) and the anterior region of the dorsal head (ptillinum, PT). CE, compound eye; OC, ocellar complex; F, frons. The asterisk marks a late-appearing *wg* expression domain around the ocelli (see also [11]). (*d,f*) Late discs from *oc2-GAL4; UAS-GFP* (*oc2 > GFP*; *d*) or *oc2-GAL4; UAS-dAxin2.28* (*oc2 > dAxin*; *f*), stained for the retinal marker Elav (white) and Ey (green). GFP expression in (*d*) is shown in magenta and marks the oc2-GAL4 expression domain. In oc*2 > Axin* discs, a duplicated eye field arises from the ocellar domain. (*e,g*) SEM images of *oc2 > GFP* (*e*) and *oc2 > Axin* adult half-heads shown at the same magnification. CEs are pseudocoloured in red. Ocelli in (*e*) are pseudocoloured in purple and the ectopic eye in (*g*) in orange. PO and F as in (*c*).

In early third-stage larvae (L3), the eye domain within the head primordium (eye-antennal imaginal disc) is subdivided into two major territories: the prospective CE expressing the Pax6 gene *eyeless* (*ey*), and the prospective dorsal head capsule, that expresses the cephalic gene *orthodenticle* (*otd*) (figure 1). *wg,* monitored by a *wg-Z* transcriptional reporter, shows a domain of strong expression within the Otd-expression territory (figure 1*a,b*). *wg* expression evolves during development so that, in the adult head, it maps to the periocular cuticle, in between the eye and the dorsal head or ‘vertex’, and to a domain anterior to the OC (figure 1*c* and [11]). To investigate the role played by *wg* signalling in the dorsal head, we targeted the Wnt-canonical signalling pathway, which is mediated by β-catenin (Armadillo, Arm; [12]), in three ways: (i) by overexpressing dAxin, an Arm-destruction complex component [13,14], (ii) by sequestering Arm to the adherens junctions through the overexpression of the intracellular moiety of E-cadherin [15], and (iii) by overexpressing a dominant-negative form of TCF (TCF^DN^), the nuclear cofactor of Arm [16]. These genetic manipulations were induced specifically in the developing dorsal head region using the strain *oc2-GAL4* driving *dAxin*, *DECad-5i* or *TCF^DN^,* respectively (see electronic supplementary material, Methods) (figure 1*d*). In all three conditions, we observed an expansion of the *ey-*expressing domain adjacent to a new field of ELAV-positive photoreceptors in discs (figure 1*f*; electronic supplementary material, figure S1). In the adults, the dorsal head cuticle (including the OC) was often transformed into CEs surrounded with periocular cuticle (figure 1*e–g*), reflecting the disc phenotype. We interpret it as a medial-to-lateral transformation of the head. The *oc2 > dAxin* (*WntKD*) genotype expressed the medial-to-lateral phenotype with the highest penetrance (28% of adults with one or two ectopic eyes, *N* = 118), and was used for further analyses.

### (b) Wnt signalling and *otd* exclude Pax6/*ey* expression from the dorsal head

**Figure 2.**
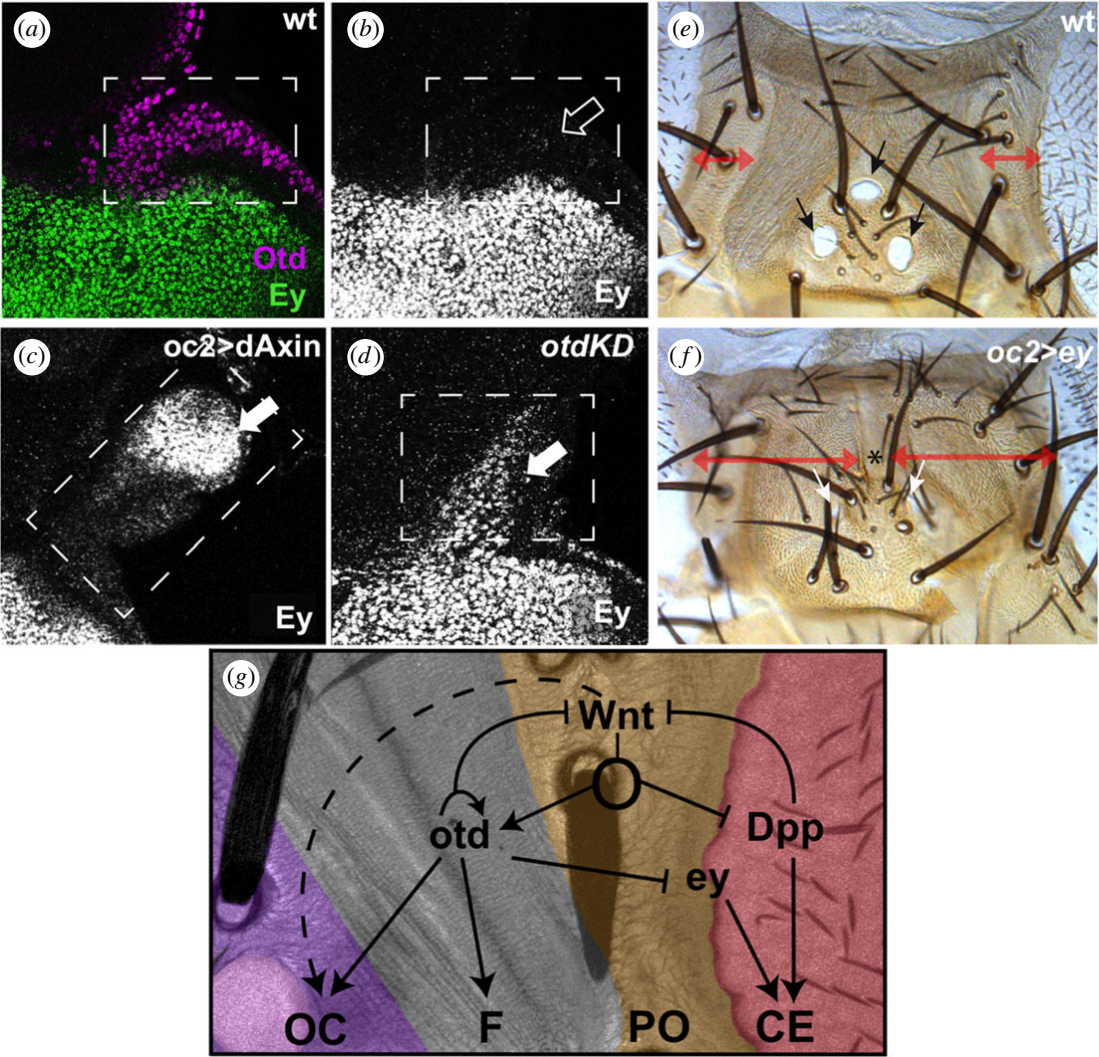
Repression of *ey* by *wg* and *otd* in the medial head. (*a–d*) Confocal images of control (‘wt’, *a*,*b*), *oc2 > dAxin* (*c*) and *oc2 > otd-RNAi (‘otdKD’, d*) discs stained for Ey (*a*–*d*) and Otd (*a*). The boxes mark the prospective ocellar region. While Ey is not expressed in the ocellar region of control discs (open arrow), Ey is derepressed in this area in both *oc2 > dAxin* and *otdKD* discs (white arrows). Derepression is stronger in *oc2 > dAxin* discs. Overexpression of Ey in the developing ocellar region (*f*; *oc2 > ey*) results in the obliteration of the ocelli and the replacement of the ridged cuticle of the head vertex by periorbital-type cuticle, characterized by bearing bristles (compare with control in ‘*e*’. Double-headed arrows mark the extent of the periorbital cuticle in ‘*e*’ and ‘*f*’). A wild-type dorsal head is shown for comparison (*e*). Arrows in (*e*) and (*f*) mark the ocelli. The lateral ocelli in (*f*) are very reduced, while the anterior ocellus is missing (*). (*g*) Medial identity of the dorsal head is imparted by Wnt signalling through Otd-dependent and Otd-independent mechanisms. The Wnt target Otd represses *ey,* which is a CE selector transcription factor. In addition, attenuation of Wnt signal leads to the derepression of *dpp. ey* and *dpp* are both necessary for further CE development. Although Otd represses *ey,* both genes are coexpressed in the periocular cuticle, where they may instruct this fate. See the main text for references.

To understand the mechanisms by which Wnt signalling controls the choice between medial and lateral head fates, we focused on key medial/lateral genetic differences and analysed how these could contribute to the action of Wnt. *otd* expression is almost complementary to that of *ey* in L3 (figure 2*a,b*) filling the prospective dorsal head. In WntKD discs, a new domain of Ey expression appeared where the ectopic dorsal eyes are to develop (figure 2*a–c*). As *otd* lies downstream of *wg* [9,11,17], we tested if *otd* could repress *ey* expression. Indeed, in discs where *otd* expression was attenuated (*oc2 > otd-RNAi,* or *otdKD) ey* expression extended medially into the ocellar region (figure 2*d*). Ectopic expression of *ey* is known to cause the respecification of fly appendages into CEs [18] so, in principle, *ey* deprepression could be causing the development of dorsal eyes in *WntKD* individuals. However, the ectopic expression of *ey* that we detected in *WntKD* and *otdKD* is unlikely to be the sole responsible of the transformation. This is because despite *ey* de-repression, there is no medial-to-lateral transformation in *otd* mutants [19] or *otdKD* [20]*.* As *ey* derepression was weaker in *otdKD* than in *WntKD* (figure 2*d,c*, respectively)*,* we tested if higher *ey* levels were capable of respecifying the dorsal head as CE by forcing *ey* expression in the ocellar region (*oc2 > ey*). This caused the obliteration of the ocelli and the expansion of the periocular cuticle, characterized by long bristles (figure 2*e,f*), but not the respecification of the medial head into CE. Therefore, *otd* expression attenuates *ey* expression in the prospective dorsal head, but the sole derepression of *ey* is insufficient to explain the medial-to-lateral transformation in *WntKD* flies. In addition, we noted that in late L3 discs, the expression domains of *otd* and *ey* overlap in a strip of cells (electronic supplementary material, figure S2) that would correspond in the adult to the periocular cuticle. As mentioned above, the overexpression of *ey* in the *otd-*expressing developing head vertex (*oc > ey*) resulted in an expansion of periocular-like cuticle (figure 2*e,f*). The converse expression of *otd* in the *ey-*domain (in *optix > otd* individuals, see electronic supplementary material, Methods) resulted in a reduced eye and extra cuticle with bristles (electronic supplementary material, figure S2*d*)—again this could be interpreted as an eye-to-periocular cuticle transformation. Therefore, the overlap of *otd* and *ey* in the disc seems to specify periocular head fate.

## 3. Discussion

The overall anatomy of the head is conserved within insects, even if its segmental composition and the genetic specification of these segments have been subject to much debate (see [3]). Recent work points to a consensus regarding the genetic underpinnings of cephalic structures. This includes the consecutive domains of Six3/optix and Otd expression at the anterior-most cephalic/brain region, and the contribution of *wg*/Wnt-1 to the specification of anterior head/ocular structures and the origin of the dorsal head, including eyes and head vertex, in the most anterior embryonic segment, the ocular/pre-antennal segment [21]. Most of these studies focused on the embryonic development of the larval head. Depending on the degree of metaboly in the species under study, conclusions on larval head development can be projected into the adult head. In *Drosophila,* an extreme holometabolous insect with a highly modified involuted larval head, these studies ought to be performed on the eye–antennal disc, the larval primordium of most structures of the adult head. However, the developmental convergence into a similar adult head in insects suggests a conservation of the genetic mechanisms specifying and patterning it, despite the intermediate diversification of the larval head in different insect groups. Therefore, it is likely that the functions performed by *wg/*Wnt-1 during the patterning of the *Drosophila* head are also widely conserved.

### (a) Early *wg/*Wnt signalling specifies medial head structures

Our results show that Wnt signalling is necessary for the specification of dorsal/medial head structures as, in its absence, the lateral fate (including eye formation) is taken by default. Indeed, forced expression of *wg,* or the activation of its pathway in the eye, leads to its transformation into head capsule-like tissue (although no ectopic ocelli, indicative of dorsal head identity, have been reported in these experiments [22]). Selection of medial head/vertex development by wg/Wnt signalling seems to proceed through a complex mechanism, because the loss of its target *otd* alone (or the concomitant gain of *ey* expression) does not recapitulate the effects of wg/Wnt signalling attenuation. We have noted that the *WntKD* disc phenotypes resemble those reported for ectopic *dpp* expression [23]. Therefore, medial head identity would be regulated by *wg* through two mechanisms: establishing an otd+/ey− domain where ocelli develop, and by preventing *dpp* expression and/or signalling (figure 2*g*; see [10,24]). Recent work by Zattara *et al*. [25] found that RNAi-mediated *otd* attenuation in *Onthophagus* beetles resulted in the development of CEs on the dorsal head. These authors acknowledge that most beetle families have lost ocelli and speculate that *otd* attenuation might have triggered the atavistic ocellar program expressed as CE [25]. As in *Drosophila* *otd* is necessary for ocellar development, this hypothesis would indicate a lack of conservation of dorsal head patterning mechanisms between coleopterans and dipterans. Alternatively, *otd* loss could result in *ey* derepression and acquisition of CE competence of the beetle dorsal head. The fact that dorsal head identity in *Drosophila* requires *wg* signalling upstream of *otd* indicates that the way *wg* signalling is wired into the gene network controlling head development differs in *Onthophagus,* which agrees with the lack of ocelli in most beetle families.

## 4. Material and methods

Targeted gene expression manipulation was carried out using the GAL4/UAS system [26] using *oc2-GAL4* as OC-specific driver line [17]. Immunofluorescence was carried out as in [9]. Additional methods are available as online electronic supplementary material.

## Supplementary Material

Supplementary Figures 1 and 2

## Data accessibility

Available as electronic supplementary material.
