# Supplementary material for: Wnt controls the medial–lateral subdivision of the Drosophila head
Source: Biol Lett. 2018 Jul 25;14(7):20180258. doi: 10.1098/rsbl.2018.0258 (PMC6083221; doi:10.1098/rsbl.2018.0258)

Methods and Supplementary Figures for:

Wnt controls the medial-lateral subdivision of the Drosophila head

M. S. Magri, MA. Domínguez-Cejudo# & F. Casares*
GEM-DMC2 María de Maeztu Excellence Unit, the CABD (CSIC-UPO-JA), 41013, Seville, SPAIN.
* Contact: fcasfer@upo.es

MATERIAL AND METHODS

***Drosophila* genetics.** Targeted gene expression manipulation was carried out using the GAL4/UAS system (*1*). *oc2-GAL4* is an ocellar complex-specific driver line (*2*). The *optix2/3-GAL4* driver ("optix-GAL4") is expressed in the undifferentiated region of the eye domain within the EAD (*3, 4*). UAS lines used were UAS-dAxinA2-28 (*5*), UAS-dTCF^{DN} (*6*), UAS-DEcad-5i (*7*), UAS-otdRNAi (*8*), UAS-ey (*9*), UAS-otd (Flybase: 0103635), UAS-GFP (*10*). The *wg* reporter *wg-Z* is described in (*11*). Larvae were raised at 29ºC.

Immunofluorescence. Primary antibodies were: guinea pig anti-Otd (1/1000; gift of Tiffany Cook, WSU Center for Molecular Medicine and Genetics, Detroit, USA), rabbit anti-Optix (1/500; gift of Francesca Pignoni, Upstate Medical University, Syracuse, USA) and guinea pig anti Hth (1/59; GP51)(12). Rat-anti-Elav (1/1000, 7E8A10) and mouse anti-Ey (1/200) were from the DSHB, University of Iowa. Anti-mouse, anti-rabbit and anti-rat secondary antibodies, conjugated with Alexa 488, 568 or 647 were from Molecular Probes. Immunofluorescence in eye imaginal discs and embryos was carried out according to standard protocols. Image acquisition was carried out using a Leica SP2 AOBS confocal microscope. Images were processed with Photoshop CS5 (Adobe).

X-gal histochemistry. Discs of *wg-Z* larvae were dissected in cold PBS and fixed in 0.5% glutaraldehyde for 15 min on ice. X-gal staining was performed in 1× PBS, 0,1% Triton X-100 (PBT), 2 mM $MgCl_2$, 5 mM $K_3Fe(CN)_6$, 5 mM $K_4Fe(CN)_6$, 1 mg/ml X-gal at 37°C. After staining, discs were washed in PBS and mounted in 80% glycerol. Adult cuticle X-gal staining was as in (*13*).

Scanning electron microscopy (SEM). Adult flies were transferred to 75% ethanol for 24 h at room temperature. Flies were dehydrated through an ethanol series (80%, 90%, 95% and twice 100%; 12–24 h each step). Flies were then air-dried and mounted onto SEM stubs covered with carbon tape and sputter-coated with gold (Edwards Six Sputter). Images were obtained using a JEOL 6460LV scanning electron microscope at the CITIUS facilities (Universidad de Sevilla).

1. A. H. Brand, N. Perrimon, Targeted gene expression as a means of altering cell fates and generating dominant phenotypes. *Development* **118**, 401-415 (1993).

2. J. Blanco, M. Seimiya, T. Pauli, H. Reichert, W. J. Gehring, Wingless and Hedgehog signaling pathways regulate orthodenticle and eyes absent during ocelli development in Drosophila. *Dev Biol* **329**, 104-115 (2009).
3. E. J. Ostrin *et al.*, Genome-wide identification of direct targets of the Drosophila retinal determination protein Eyeless. *Genome Res* **16**, 466-476 (2006).
4. M. Neto *et al.*, Nuclear receptors connect progenitor transcription factors to cell cycle control. *Scientific reports* **7**, 4845 (2017).
5. K. Willert, C. Y. Logan, A. Arora, M. Fish, R. Nusse, A Drosophila Axin homolog, Daxin, inhibits Wnt signaling. *Development* **126**, 4165-4173 (1999).
6. K. M. Cadigan, M. P. Fish, E. J. Rulifson, R. Nusse, Wingless repression of Drosophila frizzled 2 expression shapes the Wingless morphogen gradient in the wing. *Cell* **93**, 767-777 (1998).
7. B. Sanson, P. White, J. P. Vincent, Uncoupling cadherin-based adhesion from wingless signalling in Drosophila. *Nature* **383**, 627-630 (1996).
8. D. Jukam *et al.*, Opposite feedbacks in the Hippo pathway for growth control and neural fate. *Science* **342**, 1238016 (2013).
9. G. Halder, P. Callaerts, W. J. Gehring, Induction of ectopic eyes by targeted expression of the eyeless gene in Drosophila. *Science* **267**, 1788-1792 (1995).
10. J. Bessa, F. Casares, Restricted teashirt expression confers eye-specific responsiveness to Dpp and Wg signals during eye specification in Drosophila. *Development* **132**, 5011-5020 (2005).
11. J. A. Kassis, E. Noll, E. P. VanSickle, W. F. Odenwald, N. Perrimon, Altering the insertional specificity of a Drosophila transposable element. *Proc Natl Acad Sci U S A* **89**, 1919-1923 (1992).
12. F. Pichaud, F. Casares, homothorax and iroquois-C genes are required for the establishment of territories within the developing eye disc. *Mech Dev* **96**, 15-25 (2000).
13. F. Casares, R. S. Mann, A dual role for homothorax in inhibiting wing blade development and specifying proximal wing identities in Drosophila. *Development* **127**, 1499-1508 (2000).

SUPPLEMENTARY FIGURES.

Figure S1. Attenuation of the Wnt signaling pathway in the prospective head vertex causes the development of ectopic compound eyes. Confocal images of imaginal discs of the indicated genotypes (A-C). The boxed regions are shown at higher magnification in (A'-C'). Discs are stained for the retinal determination gene Eya (red) and the photoreceptor marker Elav (blue). Hth (*homotorax,* green), which marks the prospective head capsule and antenna ("a")(*12*), is used as a counterstain. In (A'-C') merged, Eya and Elav channels are shown. Red arrows point to Elav-expressing photoreceptors within the ectopic eye tissue.

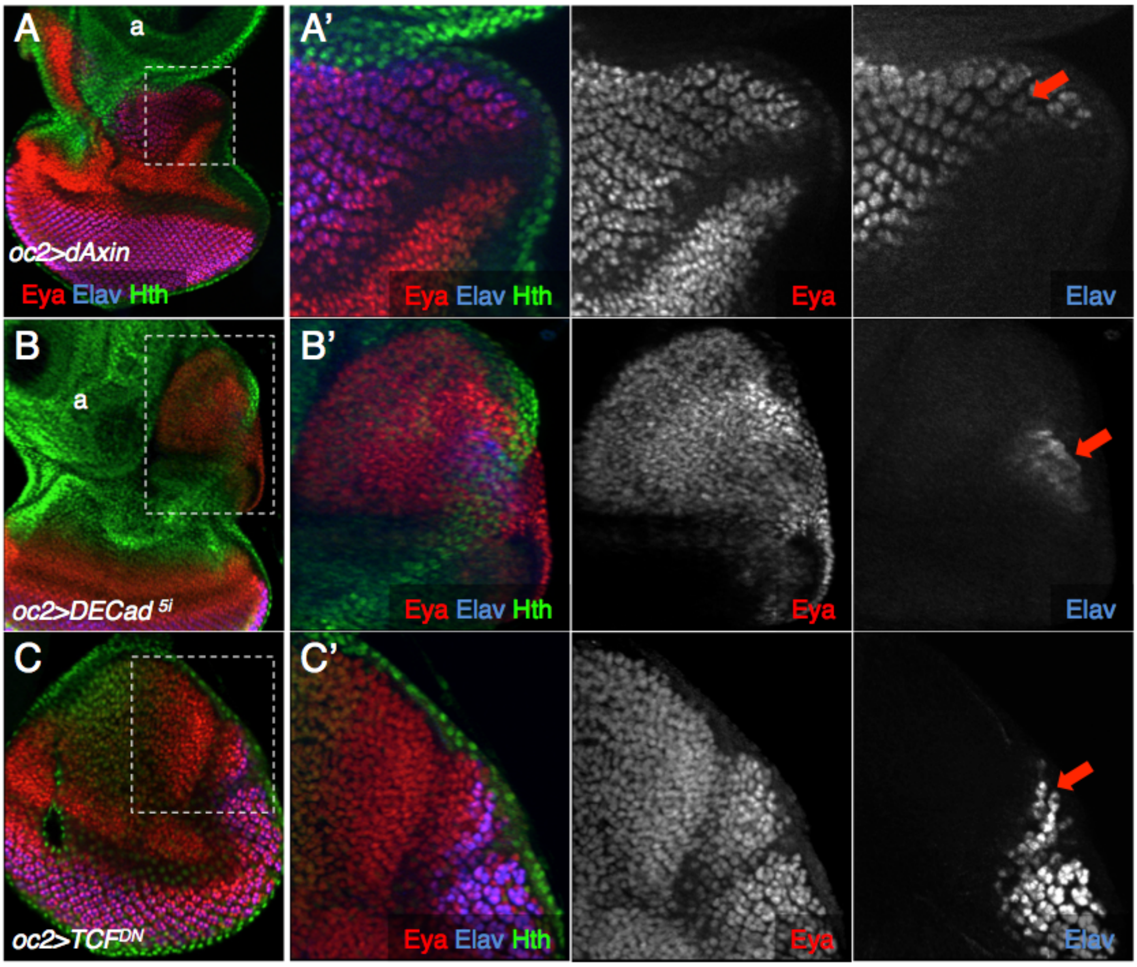

Figure S2. Ey and Otd are coexpressed in EAD and this coexpression might specifiy periorbital cuticle. (A,C,D) Dorsal views of adult heads from wildtype (A), *oc2>ey* (C) and *optix>otd* individuals (D). *oc2-GAL4* is a dorsal head specific driver, while *optix2/3-GAL4* ("optix") drives expression in the undifferentiated region of the eye primordium. The periorbital cuticle (red double-headed arrow) is extended in *oc2>ey* heads. Similarly, when *otd* is expressed in the developing eye, head-like cuticle develops at the expense of the compound eye (CE). (B) Confocal image showing the boundary between the Otd- and Ey-expressing regions in the eye-antennal disc of a late L3 wild type larva. There is a region of overlap between Otd and Ey domains that corresponds to the prospective periocular region (bracketed). Compound eye (CE) is pseudocolored in red.

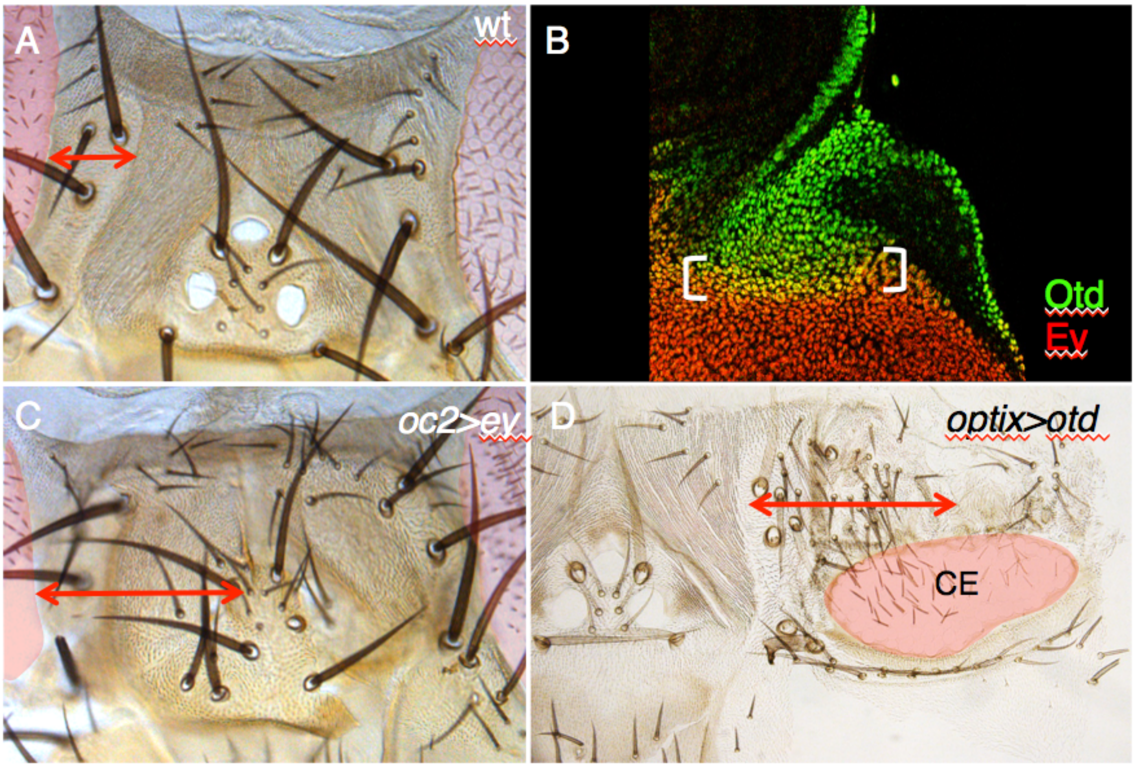

Supplement: Supplementary Figures 1 and 2 [file rsbl20180258supp1.pdf]
